# Cannabielsoin (CBE), a CBD Oxidation Product, Is a Biased CB_1_ Agonist

**DOI:** 10.3390/biomedicines12071551

**Published:** 2024-07-12

**Authors:** Mehdi Haghdoost, Scott Young, Matthew Roberts, Caitlyn Krebs, Marcel O. Bonn-Miller

**Affiliations:** 1 Nalu Bio Inc., 38 Keyes Avenue, Suite 117, San Francisco, CA 94129, USAmatthew@nalubio.com (M.R.);; 2 Charlotte’s Web, 700 Tech Court, Louisville, CO 80027, USA; scott.young@charlottesweb.com

**Keywords:** phytocannabinoids, CBD oxidation, cannabinoid receptor, biased agonist, molecular docking

## Abstract

Cannabielsoin (CBE) is primarily recognized as an oxidation byproduct of cannabidiol (CBD) and a minor mammalian metabolite of CBD. The pharmacological interactions between CBE and cannabinoid receptors remain largely unexplored, particularly with respect to cannabinoid receptor type 1 (CB_1_). The present study aimed to elucidate the interaction dynamics of CBE in relation to CB_1_ by employing cyclic adenosine monophosphate (cAMP) and β-arrestin assays to assess its role as an agonist, antagonist, and positive allosteric modulator (PAM). To our knowledge, this is the first publication to investigate CBE’s receptor activity in vitro. Our findings reveal that *S*-CBE acts as an agonist to CB_1_ with EC_50_ = 1.23 µg/mL (3.7 µM) in the cAMP assay. No agonist activity was observed in the β-arrestin assay in concentrations up to 12 µM, suggesting a noteworthy affinity towards G-protein activation and the cAMP signaling pathway. Furthermore, in silico molecular docking simulations were conducted to provide a structural basis for the interaction between CBE and CB_1_, offering insights into the molecular determinants of its receptor affinity and functional selectivity.

## 1. Introduction

**Figure 1:**
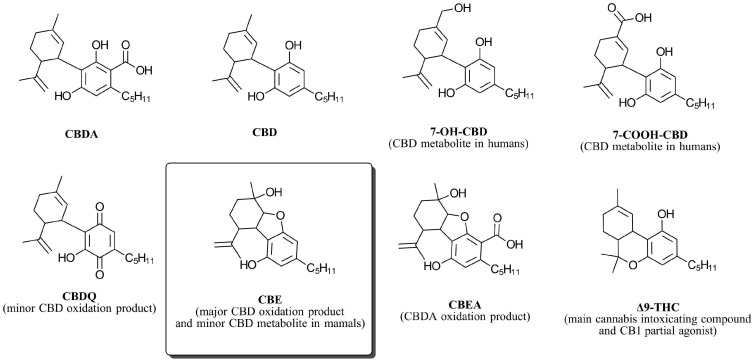
Structure of cannabidiol (CBD) and some related compounds.

Cannabidiol (CBD) (Figure 1) is a chemically sensitive molecule that is prone to isomerization, producing tetrahydrocannabinol (THC) isomers such as ∆8- and ∆9-THC when reacted with Lewis and Bronsted acids [1]. With an oxidation potential close to 1.2 volts [2], CBD is among the strongest naturally occurring antioxidants. This chemical property grants CBD oxygen sensitivity. The visual expression of CBD oxidation includes a color change from yellow to red and purple. This color change is associated with the formation of very small quantities of quinones, particularly cannabidiolquinone (CBDQ) (Figure 1) [3,4]. Due to the highly colored nature of quinones [5], measuring the oxidation level by color change is deceiving since quinones are not a major CBD oxidation product. Indeed, CBD oxidation with oxygen and other oxygen-atom-transferring oxidants (e.g., potassium peroxymonosulfate [6]) produces cannabielsoin (CBE, Figure 1) as the primary oxidation product [7].

CBE is a non-enzymatic phytocannabinoid, meaning that its natural presence in the *Cannabis sativa* L. plant has been established [8]. However, CBE is not directly produced by plant enzymes but rather by non-enzymatic transformations of other phytocannabinoids. There are two pathways for the formation of CBE, both beginning with cannabidiolic acid (CBDA), an enzymatic phytocannabinoid biosynthesized by the cannabidiolic acid synthase enzyme. In one pathway, CBDA naturally decarboxylates to produce CBD, which then oxidizes to produce CBE. In the second, CBDA is oxidized to produce cannabielsoinic acid (CBEA), which then converts to CBE upon decarboxylation. The viability of this alternative pathway is supported by the fact that CBEA is by itself a naturally occurring phytocannabinoid in *Cannabis sativa* L. [9].

CBE is also a mammalian-specific metabolite of cannabidiol. Following CBD administration to guinea pigs, the amount of CBE formed by CBD metabolism was found to be one-sixth as abundant as 7-OH-CBD [10]. Whether or not CBE is also a human metabolite is unknown. Generally, the pharmacokinetics of CBD in humans are complex but similar to guinea pigs, with 7-OH-CBD, 7-COOH-CBD (Figure 1), and their derivatives being the most prevalent metabolites of CBD in humans [11].

Being a naturally occurring oxidation product and metabolite of CBD, it is rather surprising that very little is known about the biological properties of CBE. One study reported that the body temperature of mice treated with a 10 mg/kg i.v. dose of CBE did not significantly change [10]. In this study, pretreatment with CBE also did not significantly alter the sleeping time induced by sodium pentobarbital. In contrast, the same dose of CBD significantly prolonged sleep compared to the control group. Another study reported that one of the oxidation products of CBD modulates the Wnt/β-catenin signaling pathway [12], a pathway that plays a crucial aspect in cell life and organogenesis during embryonic development [13]. The original study mischaracterized this oxidation product as CBD-epoxide. This compound was later characterized as CBE by another research group [14].

Structurally distinct from CBD-type phytocannabinoids, CBE is often classified as a separate stand-alone type [15]. However, with its three fused rings and a cyclic ether, CBE bears some resemblance to tetrahydrocannabinol (THC)-type cannabinoids. This structural similarity suggests that CBE may exhibit activity at cannabinoid receptors, particularly the type-1 receptor, CB_1_. To explore this possibility, we present the results of our investigation into the CB_1_ activity of this unique phytocannabinoid.

## 2. Materials and Methods

*S*-CBE was purchased as pure analytical standards (in acetonitrile, 1 mg/mL) from Cayman Chemicals. CP55940 (mixture of two enantiomers), AM251, and AM281 were provided by Eurofins.

### 2.1. PathHunter^®^ Arrestin Assay

For the arrestin CB_1_ agonist, antagonist, and positive allosteric modulation assays, the PathHunter^®^ β-Arrestin CHO-K1 cells, which overexpress the mouse CB_1_ receptor, were cultivated from frozen stocks using the standard protocols supplied by Eurofins, adhering to the guidelines in the cell line manual for cell cultivation (covering aspects such as culture media, supplements, and cell handling) as well as for conducting the assay and detecting signals. Cells were dispensed at a density of 5000 cells per 20 µL into white-walled, 384-well plates and then incubated at 37 °C overnight in cell plating reagent. Subsequently, a stock solution of the ligand in acetonitrile at 1 mg/mL was prepared, from which intermediate concentrations of the compound were derived through a series of ten 3-fold serial dilutions using dilution buffer in a separate dilution plate.

Agonist assay: Each dilution was prepared at a 5X concentration relative to the intended final concentration of *S*-CBE for screening. Next, 5 µL of these samples was introduced into the cells, achieving a maximum final concentration of 4 µg/mL for the *S*-CBE and 0.4% for acetonitrile (assay can tolerate up to 1% acetonitrile). Cells were incubated at 37 °C for 90 min in an atmosphere containing 5% CO_2_. To generate the assay signal, 12.5 µL of working detection solution was added to the cells, which were then left to incubate for an hour at room temperature in the dark. The signal detection was carried out using a PerkinElmer Envision instrument to measure chemiluminescence.

Antagonist assay: Each dilution was prepared at a 10X concentration relative to the intended final concentration of *S*-CBE for screening. Subsequently, 2.5 µL of these samples was introduced into the cells, achieving a maximum final concentration of 4 µg/mL for the *S*-CBE and 0.4% for acetonitrile. The assay plate was incubated at 37 °C for 30 min in an atmosphere containing 5% CO_2_. CP55940 stock solution (2.5 µL, 10X of final concentration) was added to the cells to produce a final concentration equal to EC_80_ (11.5 nM, previously calculated using agonist assay). Cells were incubated for 90 min at 37 °C and 5% CO_2_. To generate the assay signal, 12.5 µL of working detection solution was added to the cells, which were then left to incubate for an hour at room temperature in the dark. The signal detection was carried out using a PerkinElmer Envision instrument to measure chemiluminescence.

Allosteric modulation assay: Similar to antagonist assay, but the final concentration of CP55940 was 2 nM (EC_20_).

A more detailed assay protocol can be obtained from Eurofins, Luxembourg (Catalog #: 93-0471C2) [16].

### 2.2. cAMP Hunter Assay

For the cAMP CB_1_ agonist, antagonist, and positive allosteric modulation assays, the cAMP Hunter™ CHO-K1 CNR1 Gi cells, which overexpress the human CB_1_ receptor, were cultivated from frozen stocks using the standard protocols supplied by Eurofins, adhering to the guidelines in the cell line manual for cell cultivation (covering aspects such as culture media, supplements, and cell handling) as well as for conducting the assay and detecting signals. Cells were seeded in a total volume of 20 µL (10,000 cells) into white-walled, 384-well microplates and incubated at 37 °C in cell plating reagent overnight. Before adding ligands, media were aspirated from cells and replaced with 10 µL cAMP assay buffer.

Agonist assay: Stock acetonitrile solution at 1 mg/mL concentration of *S*-CBE was diluted in cAMP assay buffer to generate 3X sample containing 3X EC_80_ forskolin. Five µL of sample solution was added to cells (highest final concentration = 4 µg/mL, highest final concentration of acetonitrile = 0.4%) and incubated at 37 °C for 30 min. The assay signal was generated through incubation with 5 µL antibody solution and 20 µL working cAMP detection solution for one hour (room temperature, dark), followed by incubation with 20 μL cAMP solution A for three hours at room temperature in the dark. Microplates were read following signal generation with a PerkinElmer Envision instrument for chemiluminescent signal detection.

Antagonist assay: Stock acetonitrile solution at 1 mg/mL concentration of *S*-CBE was diluted in cAMP assay buffer to generate 6X samples. Next, 2.5 μL of 6X compound solution was added to the cells and incubated at 37 °C for 30 min. Cells were treated with 2.5 μL of the cAMP assay buffer solution containing 6X EC_80_ of (±)-CP 55,940 and 6X EC_80_ of forskolin (final (±)-CP 55,940 concentration = 1.2 nM) and incubated at 37 °C for 30 min. Similar to the agonist assay, the assay signal was generated through incubation with 5 µL antibody solution and 20 µL working cAMP detection solution for one hour (room temperature, dark), followed by incubation with 20 μL cAMP solution A for three hours at room temperature in the dark. Microplates were read following signal generation with a PerkinElmer Envision instrument for chemiluminescent signal detection.

Allosteric modulation assay: This assay is similar to the antagonist assay, but the final concentration of CP55940 was 0.2 nM (EC_20_).

A more detailed assay protocol can be obtained from Eurofins (Catalog #: 95-0071C2) [17].

### 2.3. Molecular Docking Calculations

Receptor: The Protein Data Bank obtained the crystal structure of human CB_1_ in complex with the agonist AM11542 (5XRA) [18]. The protein was prepared for docking using the UCSF Chimera Dock Prep protocol. Solvents, non-complexed ions, and the crystallized ligand were removed. Incomplete side chains were replaced using the Dunbrack 2010 rotamer library [19]. Missing hydrogens were added, and charges were assigned using AMBER ff99bsc0 [20]. Once the CB_1_ cannabinoid receptor protein model was prepared, we performed energy minimization as a prerequisite for molecular docking analysis. The minimization process was performed using UCSF Chimera with the steepest descent steps = 1000, conjugate gradient step size = 100, and step size = 0.02 Å. The binding pockets of the receptor and calculation grid were defined by AutoDockTools (center_X = −42, center_y = −162, center_z = 304).

Ligands: 3D structures of *S*-CBE, *R*-CBE, and ∆9-THC were obtained from PubChem and optimized using ChemDoodle 3D using MMFF94s force field. Flexible atomic bonds were assigned by AutoDockTools.

Calculations: Autodock Vina [21] was used for the molecular docking analysis. Grid box size of 25 × 30 × 30 and exhaustiveness of 50 were used for all molecular docking calculations. No meaningful improvement in docking scores was observed using larger exhaustiveness values. The top three best-scoring poses were selected, analyzed, and compared between the different ligands tested.

### 2.4. Statistical Analysis

Graph Pad version 10.2.2 (La Jolla, CA, USA) was utilized for the data analysis in this study. Two-way ANOVAs with Tukey tests were used to compare the significance of differences between data points across groups. A log(concentration) versus response curve with a variable slope was used to fit the data.

## 3. Results and Discussion

**Figure 2:**
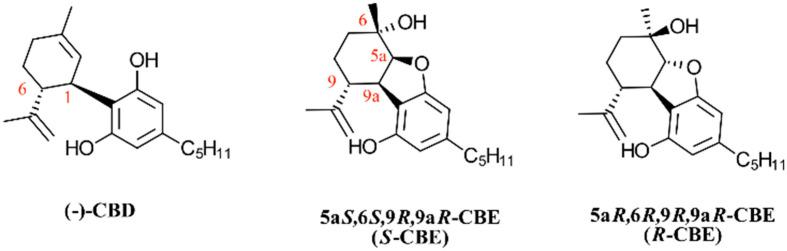
Structure of naturally occurring (−)-cannabidiol and two potential cannabielsoin (CBE) diastereomers that can be obtained from oxidation of (−)-cannabidiol. Red-colored numbers refer to carbon atom numberings.

The conversion of CBD and CBDA to CBE creates two new chiral centers in the molecule structure. With four total chiral carbons, CBE can potentially have 2^4^ = 16 optical isomers. However, starting from the naturally occurring isomer of CBD ((−)-CBD or 1*R*,6*R*-CBD), only two diastereomers can be obtained, 5a*S*,6*S*,9*R*,9a*R*- and 5a*R*,6*R*,9*R*,9a*R*-CBE, which hereby are referred to as *S*-CBE and *R*-CBE, respectively (Figure 2). Capucciati et al. have recently shown that the oxidation of (−)-CBD with dimethyldioxirane leads to the stereospecific formation of only *S*-CBE [6]. It is unknown whether the natural oxidation of CBD is also stereospecific or produces a mixture of *S* and *R* isomers. Considering that the synthetic pathway to *R*-CBE is unexplored, we only used the *S* isomer for the receptor functionality study.

The binding of phytocannabinoids to CB_1_ results in various types of receptor responses. For example, the binding of ∆9-THC to CB_1_ partially activates the receptor (agonist activity) [22]. Conversely, CBD is shown to block or dampen the biological response of CB_1_ by binding to, and blocking, the receptor activity (antagonist activity) [23]. Some studies have also demonstrated that CBD can alter the response of the CB_1_ receptor to various stimuli by binding to its allosteric site, functioning as an allosteric modulator [24]. Most cannabinoids (natural and synthetic) can also act as biased ligands of CB_1_ by inducing distinct conformational changes in the receptor, eventually translating into distinct intracellular signaling pathways through coupling to specific intracellular effector proteins [25]. For example, between arrestin and cyclic adenosine monophosphate (cAMP) pathways, WIN55,212 (a synthetic cannabinoid) is biased toward arrestin, while ∆9-THC demonstrates bias in the cAMP pathway [26]. Biased signaling has been proposed to explain the difference in the therapeutic potential and adverse effects of G-protein-coupled receptor (GPCR) ligands [27]. However, a recent study suggests that the adverse effects caused by synthetic cannabinoid agonists are mainly due to high potency and efficacy rather than their biased agonism [28].

To provide a comprehensive understanding of *S*-CBE functionality at CB_1_, we studied the agonist, antagonist, and positive allosteric modulation effects of *S*-CBE in both β-arrestin recruitment and cAMP accumulation assays.

### 3.1. β-Arrestin Recruitment Assay

Cannabinoid receptors regulate the function of various intracellular effectors, thereby governing a diverse array of biological activities [29]. Like all GPCRs, signaling cascades in CB_1_ undergo three primary modes of regulation to ensure that extracellular stimuli are translated into intracellular signals of proper magnitude and duration: (i) desensitization, rendering receptors unresponsive to sustained stimuli; (ii) internalization, involving the physical removal of receptors from the cell surface through endocytosis; and (iii) down-regulation, leading to a reduction in total cellular receptor levels [30]. The agonist-induced recruitment of β-arrestin in CB_1_ is crucial for regulating receptor activity through desensitization and internalization while facilitating signal transduction independent of G-protein activation.

**Figure 3:**
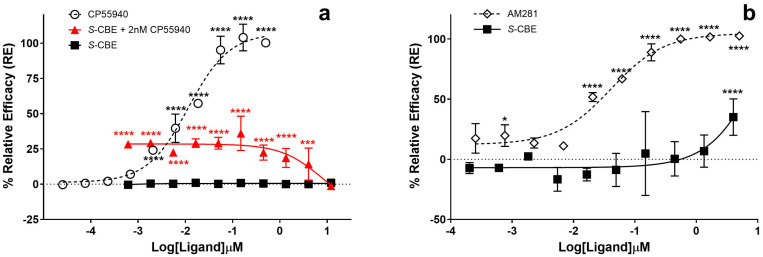
(**a**) CB_1_ agonist activity for cannabielsoin (*S*-CBE), CP55940, and co-incubation of *S*-CBE with 2 nM CP55940 (positive allosteric mode) using PathHunter^®^ arrestin assay. One hundred percent relative activity was normalized to the maximum stimulation of CP55940 and 0% relative activity to compound vehicle control. (**b**) CB_1_ antagonist activity for *S*-CBE and AM281 pretreatment on β-arrestin recruitment induced by 15 nM CP55940 using PathHunter^®^ arrestin assay. One hundred percent relative activity was normalized to the maximum inhibition of AM281 and 0% relative activity to compound vehicle control. Error bars represent the standard deviation of three independent measurements. **** *p* < 0.0001, *** *p* < 0.001, * *p* < 0.05 versus vehicle control.

When CB_1_-expressing CHO-K1 cells were treated with *S*-CBE (0.6 nM–12 µM), no significant change in β-arrestin recruitment was observed compared to vehicle control, confirming that *S*-CBE is not a CB_1_ receptor agonist in the β-arrestin pathway (Figure 3a). However, unexpected behavior was observed in the antagonist assay when the β-arrestin recruitment signal was induced by adding CP55940, a potent synthetic CB_1_ agonist, at approximately EC_80_ concentration (15 nM) after pre-treating the cells with *S*-CBE for 30 min. At the concentration of 1.34 µM and above, *S*-CBE acted as an antagonist by causing a significant reduction in the agonist activity (Figure 3b). The IC_50_ value for antagonist activity could not be calculated. Still, at the maximum tested condition (12 µM), the antagonist activity reached 35% of AM281 maximum efficiency. AM281, a potent and selective CB_1_ antagonist, produced IC_50_ = 40 nM under the same condition, indicating that the observed activity for *S*-CBE is much weaker than potent CB_1_ antagonists such as AM281 and rimonabant. Additionally, the observed antagonist activity is weaker than that of THCV isomers, ∆9-THCV and ∆8-THCV, which also show antagonist activity in the nanomolar range in the β-arrestin assay [31].

Results that aligned with the antagonist activity of *S*-CBE in β-arrestin recruitment were also obtained from the positive allosteric modulation assay, in which cells were co-incubated with a range of *S*-CBE concentrations and a fixed 2 nM concentration of CP55940 (~EC_20_). Similarly, a drop in the CP55940-induced β-arrestin signal was observed at high concentrations of *S*-CBE (Figure 3a, red line).

#### cAMP Assay

The cAMP pathway primarily regulates cellular responses through G-protein-mediated mechanisms. Traditionally recognized as a canonical signaling cascade, in the cAMP pathway, ligand binding triggers receptor conformational changes in a fashion that leads to G-protein activation, resulting in the stimulation of adenylate cyclase and subsequent production of cAMP [32].

**Figure 4:**
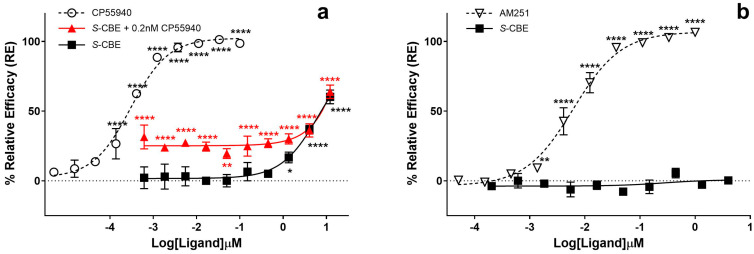
(**a**) CB_1_ agonist activity for cannabielsoin (*S*-CBE), CP55940, and co-incubation of *S*-CBE with 0.2 nM CP55940 (positive allosteric mode) using LeadHunter^®^ cAMP assay. One hundred percent relative activity was normalized to the maximum stimulation of CP55940 and 0% relative activity to compound vehicle control. (**b**) CB_1_ antagonist activity for *S*-CBE and AM251 pretreatment on cAMP accumulation induced by forskolin and 1.2 nM CP55940 using LeadHunter^®^ cAMP assay. One hundred percent relative activity was normalized to the maximum inhibition of AM251 and 0% relative activity to compound vehicle control. Error bars represent the standard deviation of three independent measurements. **** *p* < 0.0001, ** *p* < 0.01, * *p* < 0.05.

In contrast to the β-arrestin recruitment experiments, we observed clear agonist activity for *S*-CBE in the cAMP assay. When human Hunter^TM^ CHO-K1 Gi cells were treated with *S*-CBE at concentrations of 0.15 µM and above, a significant increase in chemiluminescent signal, associated with inhibiting forskolin-stimulated cAMP accumulation, was detected (Figure 4a). *S*-CBE demonstrated agonist activity with EC_50_ = 3.72 µM and, at the maximum tested concentration (12 µM), it produced ~60% E_max_ of CP55940. Under the same conditions, EC_50_ of 0.3 nM was calculated for CP55940. The EC_50_ of *S*-CBE is also significantly higher than the values reported for ∆9-THC (13 nM) using the same cell line and similar assay protocol [33]. In the positive allosteric mode, co-incubation of *S*-CBE with 0.2 nM CP55940 (EC_20_) showed an increase in the assay signal, which similarly reached 60% efficacy at the highest *S*-CBE concentration (Figure 4a, red line). No antagonist activity was observed when co-incubation happened at the EC_80_ concentration of CP55940 (1.2 nM) (Figure 4b), while the antagonist/inverse agonist of CB_1_, AM251, completely diminished the agonist signal of CP55940.

Integrating the results from β-arrestin and cAMP assays unveils an understanding of the interaction between *S*-CBE and CB_1_. In contrast to CBD, which is considered an antagonist of cannabinoid receptors, *S*-CBE emerges as a weak agonist of CB_1_, as indicated by its modest activity in the cAMP assay. However, intriguingly, *S*-CBE demonstrates a notable bias toward the cAMP pathway, showing no agonist activity in the β-arrestin assay, suggesting a distinct pharmacological profile. Its selectivity towards the cAMP pathway manifests particularly in β-arrestin assays, where *S*-CBE acts as an antagonist of non-biased agonists. Given that *S*-CBE is a metabolite and degradation product of CBD, the difference in their CB_1_ functionality prompts further investigation into how much *S*-CBE contributes to the overall biological activity ascribed to CBD.

### 3.2. In Vitro Study Limitation

A limitation of this study relates to the use of commercially available cell lines engineered to overexpress CB_1_ receptors. While these cell lines are widely used for their stability and ease of handling, they are non-human cells modified to overexpress cannabinoid receptors from humans or other mammals. Specifically, the CHO-K1 cell line used in this study was derived from Chinese hamster ovary cells engineered to overexpress non-native mouse CB_1_ receptors for the β-arrestin assay and human CB_1_ receptors for the cAMP assay. While these overexpression systems are valuable for initial screening and mechanistic studies, future studies utilizing cells that endogenously express cannabinoid receptors or validating findings in primary human cells would be beneficial to confirm the relevance and applicability of these results to human physiology.

Considering that CBE is a minor metabolite in mammals, it remains premature to ascertain whether the concentrations utilized in the in vitro assays will be clinically relevant for translation to rodent models or other species. Additional in vivo studies are required to establish the pharmacokinetic and pharmacodynamic profiles of CBE in various animal models and, ultimately, in humans. In this context, it is crucial to recognize that CBD is often administered at very high doses to achieve significant brain and plasma concentrations. For instance, a systematic review of human clinical studies by Silmore et al. [34] highlighted that the peak plasma concentration (C_max_) of CBD can exceed 1 mg/mL (3.18 mM) following a single high dose of 1.5 g.

### 3.3. In Silico Study

**Figure 5:**
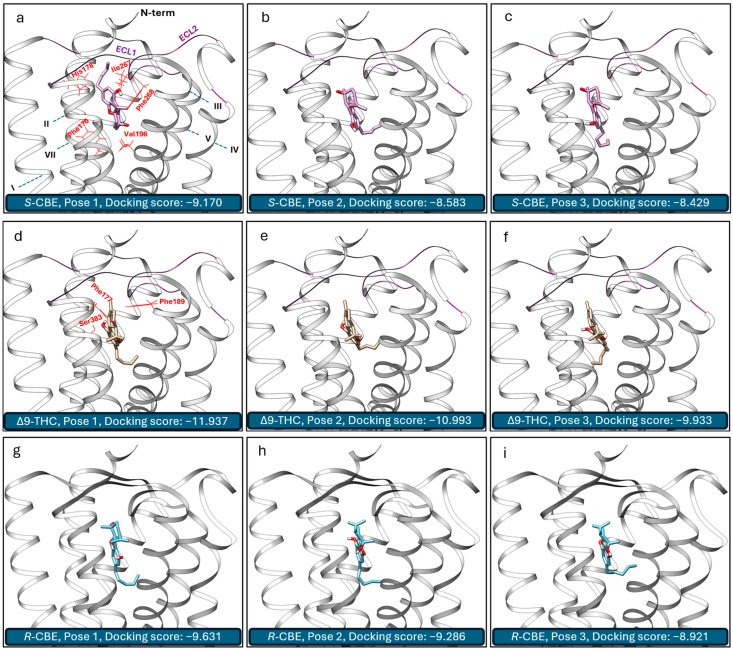
Top three energetically favorable 3D binding poses of *S*-cannabielsoin (*S*-CBE) (**a**–**c**), ∆9-tetrahydrocannabinol (∆9-THC) (**d**–**f**), and *R*-cannabielsoin (*R*-CBE) (**g**–**i**) inside the CB_1_ receptor pocket. Helices and loops are noted in (**a**). Key surrounding amino acids stabilizing the interaction of the CB_1_ receptor with the ligand (VDW overlap > 0.3) are highlighted in red in (**a**,**d**).

To enhance our comprehension of the activity of CBE at CB_1_, we studied its interactions with the active state of CB_1_ using molecular docking simulations and compared the data with that of ∆9-THC. Following our previous validation study [31], the crystal structure of human CB_1_ in complex with the agonist AM1542 (5XRA) [18] was used as the receptor structure for this study. The top three poses of *S*-CBE and ∆9-THC obtained by Autodock Vina calculation and their corresponding docking scores are presented in Figure 5. The three primary poses of ∆9-THC feature hydrogen binding between the phenolic OH group and Ser383, along with π–π interactions of the benzene ring with phenylalanines, Phe102 and Phe379. Other research groups have similarly discussed this binding pose [35]. In addition, we have recently shown that 9*R*-hexahydrocannabinol (9*R*-HHC) takes a similar pose inside the CB_1_ receptor pocket, explaining its almost identical activity to ∆9-THC [31]. The main contrast among these top three poses of ∆9-THC lies in the orientation of the pentyl group within the receptor pocket (Figure 5d–f).

During our in silico investigation into the interaction of *S*-CBE with the agonist state of CB_1_, a novel energetically favorable pose emerged. Distinct from previous poses, in this configuration, which has the best docking score, the alkyl chain diverges from the central axis of the receptor and instead occupies one of the side pockets (Figure 5d–f). Consequently, it establishes interactions with fresh lipophilic groups, notably forming strong bonds with lipophilic amino acids from extracellular loop 2 (ECL2), Phe268 and Ile267. Similar interactions have been recently reported for AMG315, a potent agonist analogue of arachidonoyl ethanolamide (AEA) [36]. *S*-CBE, however, is deprived of forming hydrogen bonds with Ser383. A previous study has shown that mutating Ser383 to alanine leads to a significant reduction in the binding of several CB_1_ ligands [27], suggesting that the lack of hydrogen bonding with Ser383 is the reason for the lower potency of *S*-CBE compared to ∆9-THC. In addition, with ∆9-THC, the agonist molecule sits on the top of the toggle switch residue of the receptor, contacting Phe200 (VDW overlap > 0.0). However, the *S*-CBE molecule sits higher in the CB_1_ binding pocket, further from the toggle switch. For example, the closest distance between Phe200 and ligand was calculated to be 3.84 and 5.72 Å for ∆9-THC and *S*-CBE, respectively. Using in silico calculations and structure-activity relationship data, it has been shown that the efficacy of CB_1_ ligands depends on their interaction with the toggle switch residues [36]. The lack of strong interaction with these residues can be considered another contributing factor to the low agonist potency and efficacy of *S*-CBE.

It is noteworthy that the second and third energetically favorable poses for *S*-CBE are similar to the ∆9-THC main agonist pose. However, the difference in the Vina docking score suggests that *S*-CBE is unlikely to take these poses inside the receptor pocket. Overall, the agonist activity of *S*-CBE at the CB_1_ receptor in a cAMP assay is significantly weaker compared to ∆9-THC, which exhibits agonist activity in the nanomolar (nM) range. This pronounced difference in activity indicates that *S*-CBE has a much lower potency in stimulating the CB_1_ receptor, a key player in mediating the psychoactive effects of cannabinoids. Consequently, our in vitro data suggest that *S*-CBE is unlikely to produce THC-like intoxicating effects at biologically relevant doses, highlighting its potential as a non-psychoactive cannabinoid in therapeutic applications.

As the synthesis of enantiopure *R*-CBE is not known, it was unable to be sourced or synthesized for inclusion in the in vitro study. In silico docking was utilized as an alternative method to predict the activity of *R*-CBE on CB_1_. Docking simulations revealed that *R*-CBE not only had a slightly higher docking score compared to *S*-CBE but also exhibited a binding pose more similar to Δ9-THC within the CB_1_ receptor pocket (Figure 5g–i). Despite this, the docking score for *R*-CBE was still significantly lower than that of Δ9-THC. These findings suggest that *R*-CBE likely possesses partial agonist activity, being more potent than *S*-CBE but still much less potent than Δ9-THC. However, it is important to note that the static nature of docking data cannot provide information regarding the potential bias of *R*-CBE towards β-arrestin or cAMP pathways. A breakthrough in *R*-CBE synthesis would enable its sourcing and facilitate in vitro studies to better understand its biological activity.

## 4. Conclusions

For the first time, *S*-CBE was discovered to be an agonist of the cannabinoid receptor CB_1_, with a significant bias toward the G-protein activation pathway. In forskolin-stimulated cAMP accumulation, *S*-CBE demonstrates agonist activity with an EC_50_ of 3.7 µM while antagonizing CP55490 in the β-arrestin recruitment experiment. This distinctive behavior underscores the complexity of *S*-CBE pharmacodynamics, hinting at its potential as a valuable tool for dissecting the signaling pathways associated with CB_1_. Based on molecular docking calculations, we concluded that the interaction of *S*-CBE with ECL2 dictates its biased agonist activity, while the lack of interaction with Ser383 and toggle switch residues is responsible for its low potency and efficacy. Further studies investigating the effects of *S*-CBE in animal models and human clinical trials could significantly enhance our understanding of its biological activity and therapeutic potential. It is important to note that this study was conducted on *S*-CBE, and future research should investigate how the other enantiomer of CBE (*R*-CBE) performs in similar assays. Finally, given CBD’s antagonist activity at the CB2 receptor, future research should compare the activities of CBE and CBD at this receptor to enhance our understanding of their distinct pharmacological profiles.

## Data Availability

The original contributions presented in the study are included in the article, further inquiries can be directed to the corresponding author.
